# Huge renal epithelioid angiomyolipoma – A case report of a giant, benign renal mass

**DOI:** 10.1016/j.ijscr.2019.12.032

**Published:** 2019-12-28

**Authors:** Mohammad Ali Ghaed, Maziar Daniali, Paniz Motaghi, Hamid Jalali Sohi

**Affiliations:** a Iran University of Medical Sciences, Urology Department, Iran; b Iran University of Medical Sciences, General Surgery Department, Iran

**Keywords:** Case report, Renal mass, Angiomyolipoma, Renal hamartoma, Epithelioid angiomyolipoma, Giant renal mass

## Abstract

**Introduction:**

Renal angiomyolipoma (also called renal hamartoma) is a benign Tumor, composed of adipose tissue, smooth muscle tissue, and blood vessels. Tumors more than 10 cm (referred to as “giant” AMLs) are infrequent and a very small number of reported cases were measured more than 20 cm. Epithelioid angiomyolipoma (EAML), is a rare variant of angiomyolipoma with malignant potential. There are less than 10 cases of huge EAML reported in English literature, that were all associated with recurrence and complications.

**Case presentation:**

Here we report a rare case of renal EAML in a 48-year-old male patient that was not associated with tuberous sclerosis. He was admitted to our hospital with a complaint of severe abdominal pain. Computer Tomography Scan (CT-scan), revealed two masses attached to the left kidney with a size of 32 × 22 × 8 cm and 20 × 10 × 3 cm. The patient underwent a radical left nephrectomy. Postoperative histopathological examination confirmed the diagnosis of an EAML. The two-year follow-up CT-scan showed no recurrence.

**Conclusion:**

Malignant EAML, although rare, does exist. Thus, besides evaluations required for ruling out the diagnosis of renal sarcomas, surgeons must follow patients after total nephrectomy for possible recurrence. Benign cases can be completely cured by surgery and do not show recurrence at the end of the follow-up period.

## Highlights

• Renal angiomyolipoma is a benign Tumor, composed of adipose tissue, smooth muscle tissue and blood vessels.
• Epithelioid angiomyolipoma (EAML), is a rare variant of angiomyolipoma with malignant potential.
• There are less than 10 cases reported as huge (larger than 10 cm) epithelioid angiomyolipoma in the English literature, all associated with recurrence and complications.
• We reported a case of benign giant epithelioid angimyolipoma without association to Tuberous Sclerosis.
• The patient was a benign case treated via surgical resection and no concurrent treatment was required to achieve great outcome.

## 1 Introduction

Typical Angiomyolipoma (AML) is a benign mesenchymal neoplasm. Epidemiological studies revealed that AML accounts for about 1 % of all resectable renal masses [1].

The incidence rate of AML is about 0.3 %–3.0 %. Tumors more than 10 cm (referred to as “giant” AMLs) are rare. Only a few reported cases, had giant tumors larger than 20 cm.

Angiomyolipoma is either find sporadically (Sporadic AML, or SAML) or in association with other conditions (such as Tuberous Sclerosis Complex Associated AML or TSCAML) [2]. The association with Tuberous Sclerosis Complex (TSC) is well known in the literature [1,3].

Immunohistochemically, AML is characterized by co-expression of melanocytic markers (HMB-45 and Mart-1/Melan A) and myoid markers (SMA and muscle-specific actin). Despite its benign behavior, it can invade the renal vascular system and involve the lymph node [1].

World Health Organization (WHO) has classified renal AML into two categories; Classical AML and Epithelioid Angiomyolipoma (EAML). The Classical AML is a benign mass composed of variable amounts of triphasic histology, a variable amount of adipose tissue, spindle and epithelioid smooth muscle tissue originating from perivascular epithelial cells and abnormal thick-walled blood vessels, despite the presence of focal nuclear pleomorphism and mitotic activity in few cases [1,[4], [5], [6]].

AML was clinically considered a benign lesion since recurrence and metastasis barely occurred in patients after surgical treatment. Nevertheless, EAML is a rare variant of AML, composed of epithelioid cells and scarce adipose tissue. EAML has been reported to pursue a more aggressive clinical course [7].

EAML is a subtype of perivascular epithelioid cell tumors (PEComas) with malignant potential. Around 160 cases of EAML are reported in the English literature until 2018 [8].

In contrast to classic AML, EAML is a potentially malignant mesenchymal neoplasm with

possible lymph node metastasis and distant metastasis [4].

EAMLs are mostly found in the kidney. They can also involve other organs including the liver, lungs, pancreas, bladder, prostate, uterus, ovary, vulva, vagina, and bone with lower incidence [7].

EAMLs are mainly composed of the human melanoma black (HMB)- 45 epithelioid cells arranged in sheets and lack adipocytes [4,8]. Based on the literature, HMB-45 can be used to differentiate between EAML and other tumors like sarcoma. It has been suggested that malignant progression of EAML may be predicted by the percentage of epithelioid cells; <10, 80–95, and 95 % epithelioid cells were associated with no, low (5 %), and high progression rates (51.5 %), respectively [9].

Since the ﬁrst description of EAML, many case reports and small series of this entity have been published. Although it is now a well-known entity, morphological criteria to differentiate EAML from renal epithelial neoplasms are not well deﬁned. EAML has no specific clinical or radiological feature, therefore the diagnosis of EAML is mostly based on pathological findings. Thus performing a biopsy and surgical resection is considered as the gold standard of diagnosis and treatment [10,11].

Surgical resection is the treatment of choice for the management of EAML. However, advances in minimally-invasive therapies and novel targeted chemotherapeutics have increased the options for the management of AML [10].

Here we report a rare case of giant renal EAML in a male Iranian patient who was diagnosed at an academic institution in Tehran. This case was benign and not associated with TSC. This project has been reported in line with the SCARE criteria [12].

## 2 Case report

Here we report a 48-year-old male case of EAML of the kidney which is a relatively newly described renal neoplasm and is closely related to the more common classic AML of the kidney and perivascular epithelioid cell neoplasm of extra-renal sites [11].

The ethical committee of our institute gave us permission for the current publication. The patient gave informed consent and is aware of this case report.

The patient arrived at our institute, complaining of severe pleuritic chest pain for approximately three months. He was not a smoker and did not consume any illicit drug or alcohol. He had never experienced urinary problems. His past medical history was unremarkable. He had no history of previous surgery.

On physical examination, a huge abdominal mass was palpated in the left flank. Incredibly, he did not feel this enormous mass earlier which may suggest the rapid growth of the lesion.

**Fig. 1:**
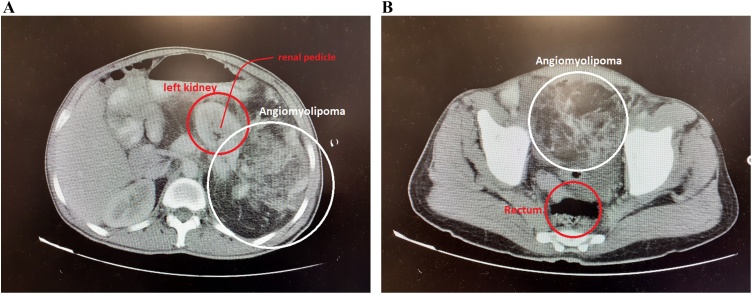
Computed Tomography Scan at the time of diagnosis. A- representing the origin of larger tumor from left renal pedicle B- representing the huge mass extended to pelvis

There were no abnormal findings in laboratory studies (Including complete blood count, urine analysis and stool examination) except for a slightly elevated Erythrocyte Sedimentation Rate (ESR). Renal and liver function tests were within normal limits. He was admitted for further diagnostic evaluations. The chest Computed Tomography scan (CT-scan) revealed a massive pleural effusion in the left hemithorax. Abdominopelvic CT-scan showed two infrarenal heterogeneous masses arising from the medial pole of the left kidney with significantly increased vascularity. Both masses contained adipose tissue, which was suggestive of either AML or a well-differentiated liposarcoma (Fig. 1).

Purified Protein Derivatives (PPD) skin test and cytopathologic analysis of pleural fluid were done in order to rule out tuberculosis and malignancy respectively. The aspirated fluid only contained inflammatory materials.

**Fig. 2:**
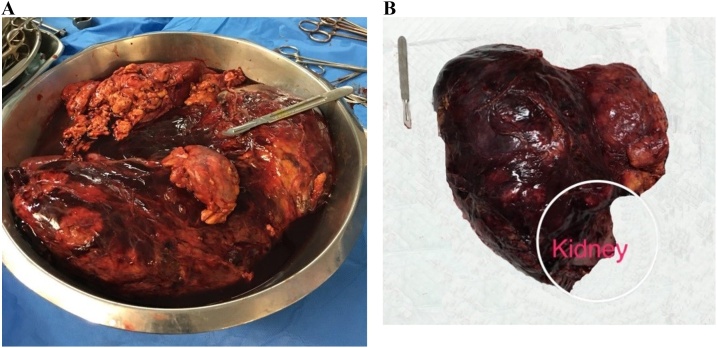
(a and b) Giant renal angiomyolipoma after resection.

Then, he underwent a radical left nephrectomy which is the treatment of choice for huge AML of the kidney based on the literature. Surgery was performed by an experienced urologist, under general anesthesia, and via a midline incision. After exploring the abdominal cavity, two huge masses attached to the middle pole of the left kidney were observed (Fig. 2). Massive intra-operative bleeding led us to infuse one unit of homologous cross-matched packed cells. Masses were measuring 32*22*8 cm and 2*1*0.5 cm, with lobulated surfaces and covered by a thin capsule. On cut sections, both masses contained soft, creamy yellow tissue comprising a growing adipose tissue. The smaller mass was separately located into perinephric fat while the greater one, was invaded renal sinus fat. After the operation, he was admitted to the Intensive Care Unit for 4 days and then to the urology ward for a week. The post-operative period was uneventful and the scar healed perfectly.

**Fig. 3:**
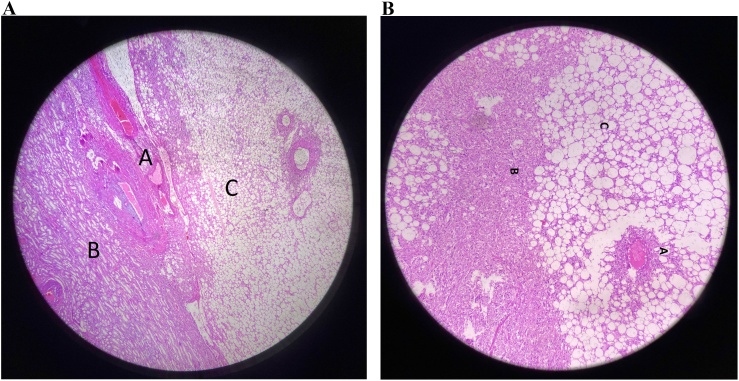
(a and b) High power view of specimen sent for histopathologic evaluations, representing all three types of consisted tissues (A: blood vessel tissue, B: smooth muscle tissue, C: adipose tissue).

The specimen was sent for histopathologic evaluations. The larger mass contained necrotic tissue and atypical epithelioid cells with the mitotic rate of 0–1 IN 10 HPF. The atypical mitotic figure was not identified. These findings completed the gross characteristics to confirm the diagnosis of EAML, possibly associated with tuberous sclerosis. (Fig. 3)

Lastly, by the consult of an expert dermatologist, the association with Tuberous Sclerosis Complex (TSC) which presents with multi-system progressive tumors, intractable epilepsy, and mental retardation, was ruled out. He had no skin lesion suggestive of sclerotic changes or a history of neurological manifestations of TSCs.

**Fig. 4:**
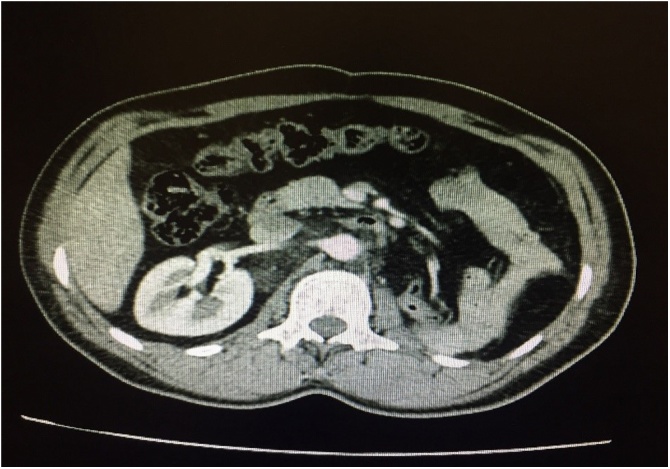
Follow-up Computed Tomography scan.

No specific concurrent treatment was applied for him except antibiotic (1 g of Cefazolin every 6 h) and analgesia (when required) for two weeks postoperatively. He was under our close observation during his stay at the hospital. Renal Function Test and Complete blood count were evaluated every other month. His abdominal pain was completely relieved after the surgery. At a two-year follow-up by CT-scan. There has been neither metastasis nor recurrence during the 24-month period since resection (Fig. 4).

## 3 Discussion

EAML in contrast to the classic AML (which is benign), may be associated with distant metastasis and local recurrence. Malignant behavior and metastasis to lymph nodes, liver, lungs or spine has been reported in approximately one-third of cases of EAML [13].

Over a decade, the diagnosis and management of renal AMLs have been discussed in the literature. The deﬁnitive diagnosis of primary renal EAML needs both histochemical and pathological evaluations since there are no definite clinicopathological criteria [9].

Radiologic characteristics of EAML cannot help in making a definite diagnosis. Some of the imaging manifestations of EAML are similar to renal carcinomas. However, the use of immunohistochemical examinations has to be performed to achieve the exact diagnosis.

On CT-scan, soft tissue lesion with higher density (compared to normal renal parenchyma) and enhancement pattern of “Rapid wash-in and slow wash-out” are suggestive of EAML.

On Magnetic Resonance Imaging (MRI), EAML has a low-to-high T1WI signal (due to acute hemorrhage), low T2WI signal (with diffusion restriction due to hypercellularity of mass), distinct margins, and noticeable heterogeneous enhancement but lacks adipose tissue.

On Ultrasonography, EAML is seen as a hypoechoic lesion with heterogeneous peak enhancement that may have a pseudo capsule [10].

In our current case, the imaging studies revealed two infra-renal heterogeneous masses arising from the medial pole of the left kidney with significantly increased vascularity. Both lesions had characteristics of adipose tissue which suggest the diagnosis of either AML or a well-differentiated liposarcoma.

Despite the benign behavior of most AMLs, few researchers reported that some lesions may be malignant when first observed or may later undergo malignant change and need immediate supervision. The issue was that later histopathologic evaluations of three previously reported cases of malignant AML without association with tuberous sclerosis suspected the primary diagnosis. Thus, some pathologists, have questioned the existence of a malignant EAML, when the diagnosis is not confirmed by immunohistochemical results [5].

Recently, several Cases of malignant EAML with metastasis to the liver, lung, and bone have been reported. The similarity in the morphology of epithelioid cells and renal cell carcinoma (RCC) has made the diagnosis of EAML challenging [8].

EAML is the malignant variation of renal AML, that can be distinguished by containing a large number of human melanoma black (HMB)- 45 epithelioid cells and lacking adipocytes [8].

We believe a malignant EAML, although rare, does exist. Many of the previously reported cases of malignant AML lack the fat cells and abnormal blood vessels that are seen in the classic AML. Some of them were primarily misdiagnosed with renal cell carcinomas before the immunohistochemical evaluations confirm the diagnosis of AML.

The small number of detected AMLs with malignant behavior makes it impossible to define a criterion for distinguishing benign from malignant EAMLs. Even though the significance of necrosis is under question since two of the researches designed by Delgado et al. [14] and Eble JN [15], on prognostic stratification of EAMLs did not support the role of necrosis in discriminating benign and malignant lesions.

In another study performed by Lee et al. [16] 587 cases of renal AMLs (SAML: 87.4 %, TSCAML: 8.7 % and EAML: 3.9 %) were retrospectively analyzed. They stated that most of their AML patients were asymptomatic. The most common symptoms were flank and abdominal pain. They also compared these three kinds of AMLs in terms of tumor size and complications and a significant difference in tumor size between the AMLs was found. In their report, the median size of SAML, TSCAML, EAML were recorded as 4.7, 2.7, 10.5 cm respectively.

Our case had 2 SAMLs with a size of 2*1*0.5 cm and 32*22*8 cm. The greater mass was notably larger than the previously reported cases. Interestingly that huge mass remained asymptomatic and did not develop any complication till enlarged to reach that size [16].

De Bree et al. reported a case of a huge renal EAML with a maximum diameter of 13 cm in the area of the resected 17 cm kidney, but their case in contrast to ours had hepatic and peritoneal metastases at the time of diagnosis [17].

Guan et al. also reported a co-existence of AML and angiosarcoma in a 64-year-old woman who was presented with a progressively enlarging mass in the left abdomen. The tumor CT-scan revealed an 18 cm × 11 cm mass in the left posterior renal fascia. Immunohistochemical examinations confirmed the diagnosis. Their reported tumor was comparable to our case, in terms of size, while, that primary renal angiosarcoma was possibly arisen in a pre-existing AML of the kidney and showed a malignant behavior [18].

Similarly, Espinosa et al. reported a case with recurrence of sporadic renal EAML in a 34-year-old patient, who underwent radical nephrectomy after diagnosis of a renal mass of 10 × 12 cm. This lesion had characteristics suggesting a poor prognosis (including size > 7 cm, vascular and renal sinus invasion, necrosis, and severe atypia). After nephrectomy, the patient did not receive adjuvant chemotherapy. Seven months after the surgery their patient developed 3 liver metastases. Their finding supports the rare, but possible malignant behavior of renal EAML. Thus, they recommended a long term follow-up after surgical resection of EAMLs [19].

## 4 Prognostic factors

Three recent studies designed by Brimo et al. [20], Nese et al. [21] and Park et al. [1] have presented prognostic factors in risk stratification of renal EAML in adult patients

The indicated unanimous factors associated with poor prognosis are namely, severe cytological, extent of nuclear atypia (more than 70 % atypical epithelioid cells), larger epithelioid cell component, presence of more than 2 mitoses per 10 high power fields, association with TSC or concurrent AML, tumor size of larger than 7 cm, lymphovascular invasion, extra-renal extension, renal vein involvement, and carcinoma-like growth patterns.

## 5 Treatment approach

Folpe, A.L. et al. [22] have been classified PEComa into three categories of benign, uncertain malignant potential and malignant based on some empirical criteria (such as tumor size, rate of mitosis, high or non-high nuclear grade, cellularity, presence of inﬁltration, necrosis or vascular invasion) to help surgeons choose the best approach for treatment.

Due to the malignant potential of renal EAML, the treatment of choice is complete surgical resection. Based on our review of the literature, all cases have remained asymptomatic after surgical management and there were no reports of recurrence after sparing nephrectomy or embolization [7].

Sometimes the surgery should be performed in combination with target therapy (m-TOR inhibitors). however, the definite effects of this therapy have not been confirmed yet [5,23]. Selective embolization and ablative therapy (such as radiofrequency and cryoablation) are other treatment options for AML which can be used during pregnancy or hemorrhage. Some researchers suggested that systemic chemotherapy does not help in achieving a satisfactory outcome in metastatic EAML [10].

The tumor was excised and the margins were free of malignancy. The patient was doing well postoperatively. We did not recommend advanced chemotherapy after the surgery. Two years later, no local recurrence and distant metastasis were found in the follow-up CT-scan.

The limitation of our study was that our patient did not accompany a further follow-up visit as he was satisfied with the outcome.

## 6 Conclusion

We believe, EAML has the potential for aggressive behavior and metastasis, so the diagnosis must be confirmed by pathological and immunohistochemical examinations when we find characteristics of this rare mesenchymal mass on the CT-scan. Considering the malignant potential of renal EAML, a long-term follow-up is required after the surgery. Our case was unique in that this patient had a sporadic giant Angimyolipoma with epithelioid content which was not associated with the TSC at the time of diagnosis. However, because of the radiologic features that EAML and malignant renal lesions have in common, renal carcinoma should be considered as an important differential diagnosis. Our patient had an uneventful follow-up period without recurrence.

## Conflicts of interest

This article has no conflict of interest.

## Funding

We had no source of funding

## Ethical approval

The ethical committee of Urology research centre at Iran University of Medical Sciences is aware of current publication and gave permission.

## Consent

Our patient gave us informed consent after making sure his information is published anonymously

## Author contribution

- Mohammad Ali Ghaed – Diagnosis and surgery – supervising this report
- Maziar Daniali – Review of articles and writing manuscript
- Paniz Motaghi – Review of articles and writing manuscript
- Hamid Jalali Solhi – performing surgery and diagnosis

## Registration of research studies

This study is not a clinical trial, thus has not been registered.

## Guarantor

Dr. Mohammad Ali Ghaed, the first author.

## Provenance and peer review

Not commissioned, externally peer-reviewed
